# On the Use of Phosphate of Ammonia in Rheumatism

**Published:** 1846-07

**Authors:** H. Hartshorne

**Affiliations:** Resident Physician, Pennsylvania Hospital


### MEDICAL EXAMINER,

AND

### RECORD OF MEDICAL SCIENCE.

NEW SERIES.—No. XIX.—JULY, 1846.

---

### ORIGINAL COMMUNICATIONS.

*On the Use of Phosphate of Ammonia in Rheumatism.* By H. Hartshorne, M. D., Resident Physician, Pennsylvania Hospital.

Since the publication of Dr. Buckler's article on this subject in the American Journal of Medical Sciences, considerable notice has been taken of it, and with various results in the opinion of different practitioners.

In the Examiner for May, of the present year, a communication appeared from Dr. Voigt, mentioning the use of it in *three grain* doses, with apparently disadvantageous effects upon the digestive apparatus. The experience of the Pennsylvania Hospital, in Dr. Pepper's trial of the medicine as detailed below, is entirely different from this. The smallest dose given to an adult here was ten grains, and in several it reached 30 grains, continued three times daily for a number of days. One of the latter cases required also at times an additional laxative. In two or three cases it did, in twenty grain doses, disorder the alimentary canal; but it appears to me impossible to expect effects of any kind from less than five or ten grains. In these quantities Dr. Ruschenberger (vide last month's Examiner) has tried it with very little benefit.

The powers of the remedy were fairly tested in the Pennsylvania Hospital,—and the result may be deduced from a short statement of each case. It will be seen that it has proved no

*pecific*, although several of the cases recovered. Additional reatment was used generally,—as Dover's powder at night, the warm bath, cupping, blisters dressed with morphia, and mercurial ointment combined with narcotics, veratria ointment, &c. These may perhaps claim much of the credit of the cures, which were mostly very slow.

James Burns, aged 32, admitted 3d mo. 14th, was put on ammon. phosph. gr. xx., aq. cinnamom. f℥ss, 3d mo. 17th, being three days after admission. He had in two of these days been taking colchicum and magnesia. He was discharged cured, 4th mo. 9th.

James Stewart, admitted 2d mo. 5th, aged 22. Both his arms and both knees were affected. Put on ammon. phosph. gr. xx., with aq. cinnam., 3d mo. 17th, forty-two days after admission. He had in this time been treated first with calomel and Dover's powder, and afterwards with colchicum and magnesia. On the 1st of 4th mo. he relapsed, after having been much better; a blister was put on the painful shoulder, and, when healed up, renewed. This seemed much to quicken his improvement; a third blister was sprinkled with morph. acet. gr. j. p. g. acac. gr. x., which still farther lessened his pain. 4th mo. 15th,—he has little or no pain, his arm is but slightly stiff, and the medicine has been stopped.

Abraham Shepherd, age 51, admitted 3d mo. 16th. One arm only affected, but this made powerless by the attack. He was put upon ammon. phosph. gr. xx. as above, on the 21st of 3d mo., being five days after admission, he having in that time taken first p. ipecac. et opii gr. iv. ter die, and afterwards vin. colch. rad. with magnesia for a short time. By the 5th of 4th mo. he had very little pain, except on motion of the arm affected at the shoulder; but some stiffness is noted as yet (4th mo. 16th) hanging on; treated with passive motion and electricity. About the 8th of 4th mo., however, he complained of diarrhœa, pain and rumbling in his belly, vomiting and loss of appetite. The remedy was then stopped, and in three or four days these symptoms disappeared.

William Curran, aged 9, admitted 3d mo. 21st. Has considerable enlargement of heart; pulse rapid, loud bellows sound. This heart disease has continued probably for some months. His arms and legs were also affected with rheumatic symptoms up to 3d mo. 22. He was then put on ammon. phosph. gr. v., aq. cinnam. ℥ss three times a day. It was suspended about 4th mo. 8th; his articular rheumatism had, before that, entirely left him, though the chronic disease of the heart remained; improved by digitalis and moderated diet.

Thomas Quin, age 46, admitted 3d mo. 25th,—limbs affected with rheumatism. Began the am. phosph. at once, using at same time, as did most of the others, the Dover's powder at night, and warm bath every other night. 4th mo. 1st he had a blister applied.

4th mo. 11th he went out, *cured.* But on the 18th of the following month he applied to be readmitted, saying that it had returned in his limbs as bad as ever. This, of course, might occur after any treatment, *specific* or not.

Leonard Kretzner, admitted 3d mo. 28th, age 38, limbs considerably affected. Put on ammon. phosph. 3d mo. 30th, after one or two doses of colchicum and magnesia. 4th mo. 3d he had another dose of the same mixture, and this, with the warm bath every other night, was all the adjunct treatment. He was discharged cured, 4th mo. 9th, having been nearly well for a week or two.

——— Cochran, had been for some months under treatment with mercury, &c., in the house, for visceral derangement. His rheumatism presented itself as a fresh acute attack. He was treated for three days with the phosphate of ammonia without any effect; the tumefaction, redness, pain and fever not yielding, and his bowels not being kept open by first gr. xx. and then gr. xxx. of the salt, it was stopped, and under colchicum and magnesia, twenty drops of the wine of the former with a scruple of the latter in mintwater, he gained rapid and permanent relief from all rheumatic symptoms.

Henry Pillsbury, age 30, captain of a vessel, admitted 4th mo. 20th. He was first bled, and treated with colchicum for about a week; then put on the use of hyd. chlor. mit. gr. j., p. opii gr. $\frac{1}{4}$, three times a day, being purged every few days with Scudamore's mixture of colchicum. He had taken thirty grains of calomel before his mouth was touched, and then was little better than at first.

5th mo. 3d, takes t. colch. rad. gtt. xx., magnes. gr. x. ter die, and has veratria ointment, twenty grains to the ounce, rubbed over his hands twice a day.

5th mo. 5th. Very little improvement. Begins now with ammon. phosph. gr. xx. aq. cinnam. ℥ss ter die. In a day or two he commenced also using the warm bath at night. He takes the Dover's powder at bed time.

8th. Is better. Bowels not open to-day. Increase ammon. phosph. to gr. xxx. ter die.

12th. Little change. Blister to affected foot, dressed with morph. acet. gr. j., p. gum. acac. gr. x. Bowels not open to-day by the phosphate. ℞. Mist. colch. Scud. statim.

13th. Apply to hands ung. hyd. fort. ℥j, ext. hyoscyam. ℨss.

14th. Ditto to knee. He is now improving.

15th. Thinks himself better, except his hands. Continue ammon. phosph., p. ipecac. et opii nocte, and warm bath.

16th. Hands better.

17th. No pain, though his knuckles are still swollen.

18th. Went out to-day, and says we may put him down cured.

Margaret Murray, age 22, admitted 3d mo. 5th. Limbs affected. Put on phosphate of ammonia, twenty gr. doses, 3d mo. 17th, having for eleven days taken the colchicum and magnesia. She was also cupped along the spine. By the beginning of 4th mo. the pains were leaving her limbs, and soon were transferred entirely to the face, constituting a rheumato-neuralgic affection of the left eye and upper jaw. Belladonna, camphor, &c., were applied, and carbonate of iron given internally for this, the ammon. phosph. being suspended She went out 4th mo. 9th, pretty much cured, and entirely so of the rheumatism of the limbs for which she entered.

Bridget Halfpenny, aged 40, admitted 3d mo. 16th. Put on phosphate of ammonia as above, 3d mo. 19th, three days after admission, having been taking colchicum and magnesia in that time.

She was obliged to suspend the phosphate two or three times on account of diarrhœa with pain which it caused, but went out 4th mo. 11th, having been quite well for a week.

In all these cases, twenty and thirty grain doses of the salt produced some laxative effect upon the bowels,—in one a painful diarrhœa; and in another, diarrhœa, vomiting and indigestion, were brought on after using twenty grains thrice daily for several days. Two patients had very little effect produced upon their bowels. No increase of the urine was, on inquiry, ascertained in any of them.

The phosphate has been used in a dozen or more cases by my father, Dr. Joseph Hartshorne, with such advantage as inclines him to think it may be a valuable addition to our means of treatment of rheumatism, though by no means as a specific remedy.

In the tardiness of cure, and repeated failure among the cases above reported, we see the proof that it has no such peculiarity or certainty of action; and that the plausible theory of Dr. Buckler is as yet unconfirmed. It is a question whether now, if ever, we can hope so clearly to unriddle the subtle chemistry of the animal fluids. But the known acid condition of the secretions in rheumatism, makes alkalies and alkaline salts a natural indication; laxatives are also called for; and this medicine has both these qualities. The degree of success with it, or at least the number of *coincident recoveries*, is also enough to justify more extended trials, although there is nothing as yet to induce us to suppose it can supersede the colchicum.

